# The pain alarm response - an example of how conscious awareness shapes pain perception

**DOI:** 10.1038/s41598-019-48903-w

**Published:** 2019-08-28

**Authors:** Moa Pontén, Jens Fust, Paolo D’Onofrio, Rick van Dorp, Linda Sunnergård, Michael Ingre, John Axelsson, Karin Jensen

**Affiliations:** 1 Karolinska Institutet, Department of Clinical Neuroscience, Stockholm, Sweden; 2 Stress Research Institute, Stockholm University, Stockholm, Sweden; 3 Institute for Globally Distributed Open Research and Education (IGDORE), Stockholm, Sweden

**Keywords:** Habituation, Human behaviour

## Abstract

Pain is subjective and largely shaped by context, yet, little is known about the boundaries for such influences, in particular in relation to conscious awareness. Here, we investigated processing of noxious stimuli during sleep. Four experiments were performed where participants (n = 114) were exposed to repetitions of noxious heat, either when awake or during sleep. A test-phase followed where participants were awake and exposed to painful stimuli and asked to rate pain. Two control experiments included only the test-phase, without any prior pain exposures. Participants in the awake condition rated all test-phase stimuli the same. Conversely, participants who had been sleeping, and thus unaware of getting noxious heat, displayed heightened pain during the first part of the test-phase. This heightened reaction to noxious stimuli—a pain alarm response—was further pronounced in the control conditions where participants were naïve to noxious heat. Results suggest that the pain alarm response is partly dependent on conscious awareness.

## Introduction

The outside world is an uncertain place and evolutionary history shows that the ability to generate adaptive responses to threat is essential for survival^1^. This requires a flexible system where threatening stimuli are evaluated and prioritized in relation to their context, and followed by behaviors that fit that particular situation^1,2^. The same threatening stimuli will thus generate numerous different behavioral responses. One striking example is the tendency to react with heightened responses to novel stimuli that will help direct attention to new sensations. In an adaptive manner, this surprise effect is usually followed by habituation to subsequent stimuli if the situation renders no reason for alarm or continued vigilance. Habituation represents a basic form of learning that is found in all species, even in single-cell organisms^3^, and is essential for reducing the influence from irrelevant contextual stimuli and to focus on stimuli that matters.

Painful stimuli represent major threats since they may be associated with harm to our physical condition^2^. Yet, the intensity of a painful stimulus is not linear to the subjective painful experience^4,5^. This means that there is flexibility in the relationship between the nociceptive input and the perceived state of the body, which enables shaping of the painful experience in a way that facilitates adaptive behaviors^6,7^.

The novelty of a painful stimulus is one factor that shapes the painful experience. Novelty often results in heightened pain responses, sometimes referred to as a “surprise effect”^8^. This surprise effect, or pain alarm response, is a well-known artifact in experimental pain research and is often controlled for in experimental procedures as it introduces variability in pain ratings. Here, the pain alarm response is not treated as an artifact but studied in itself as a salience component of basic pain processing.

Even if it is well established that pain responses are shaped by contextual and cognitive factors the boundaries for such influences are unclear, in particular in relation to conscious awareness. In this novel experimental approach, we investigated the role of conscious awareness on pain processing by comparing the pain alarm response in participants who were either aware or unaware of prior pain (as they were either awake or asleep during noxious exposures). We hypothesized that participants who were awake during noxious stimuli would have no initial pain alarm response during subsequent pain testing. Those who were unaware of receiving noxious stimuli, on the other hand, were hypothesized to display similar pain alarm response as participants who were not exposed to any noxious stimuli prior to the test-phase.

## Results

A total of 114 healthy participants (67 women, mean age 27.3 ± 8.3), were exposed to repetitions of noxious heat onto their leg either when awake or during sleep. Two control experiments included naïve participants that did not receive any noxious stimuli prior to the test-phase. 95 participants were included in the final analysis (58 women, mean age 27,2 ± 8,7). Nineteen participants were excluded due to remembering the noxious exposures and one participant was excluded due to incomplete data.

There was no significant difference in baseline characteristics on pain threshold and age between the four experimental groups, see Table 1. A mixed effects exponential growth curve model was used to assess the pain alarm response. Overall in this study, there was a significant pain alarm response, as pain ratings were initially high and then rapidly normalized to stable ratings of perceived pain (*p* < 0.001), representing the true, or latent, pain response (i.e. the asymptote). For detailed descriptions, see Table 2.

**Table 1:** Participant characteristics.

Experimental condition	Awake	Sleep	Naïve_hi_	Naïve_lo_	Welch’s ANOVA
Number of participants	n = 24	n = 30	n = 32	n = 28	
Age	27.7 ± 8.5	27.2 ± 7.4	26.7 ± 8.4	27.9 ± 9.2	*P* = 0.954
Male/female Ratio	50/50	50/50	34/66	32/68	
Pain threshold (°C)	39.9 ± 2.4	39.8 ± 2.0	40.9 ± 2.7	41.3 ± 2.8	*P* = 0.091
Maximum pain (°C)	48.4 ± 1.0	46.8 ± 2.4	48.5 ± 0.8	48.7 ± 0.9	*P* = 0.003

Pain threshold refers to the first temperature rated > 0 during pain calibration, using a 0–100 Numeric Rating Scale (NRS). Maximum pain refers to the first temperature rated >60 NRS during calibration. All values are given as Means ± Standard Deviation (SD) except for the Male/Female ratio, which is given in %.

**Table 2:** Mixed effects exponential growth curve model, predicting rated pain from number of prior stimuli.

Fixed effects	Estimate	SE	p-value
*Decline (g)*	−0.51	0.04	0.000
***Alarm response (r)***			
Naïve_hi_	15.18	0.97	0.000
Naïve_lo_	16.62	1.03	0.000
Sleep	6.86	1.65	0.000
Awake	−1.38	1.11	0.216
***Asymptote (a)***			
Naïve_hi_	22.88	0.46	0.000
Naïve_lo_	11.46	0.50	0.000
Sleep	13.08	0.70	0.000
Awake	10.89	0.48	0.000
**Random effects**	**SD**	**Correlation**	
Asymptote (η)	13.04		
Alarm response (ξ)	16.27	−0.48	
Residual (ε)	5.37		

Note. The model is based on equation 1, but with fixed effects of the asymptote (a) and alarm response (r) calculated separately for each group (Naïve_hi_ (n = 32), Naïve_lo_ (n = 28), Sleep (n = 11) and Awake (n = 24). Random effects describe the standard deviation of subject variation around the asymptote (η) and the alarm response (ξ) in addition to the residual (ε). Overall there is a significant alarm response in naïvelo (*p* < 0,001), naïve high (*p* < 0,001) and sleep condition (*p* < 0.001). No significant pain alarm response in the awake condition (*p* = 0.216).

**Figure 1:**
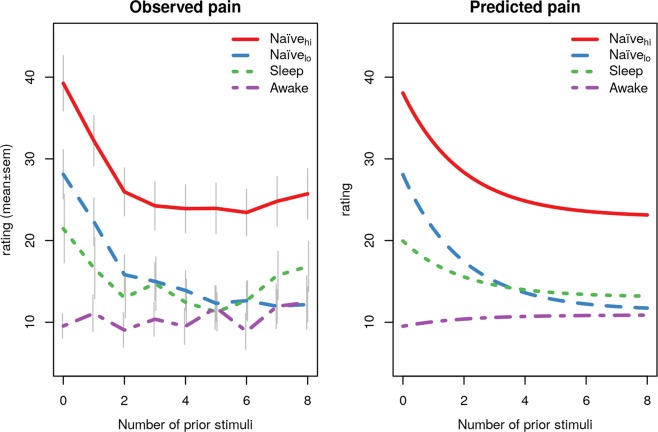
Pain alarm responses during pain testing. Graphs represent observed and predicted pain in the four experimental groups. The left panel shows observed mean ± standard error of the mean for the four groups, and the right panel shows the (fixed effect) predicted pain in each group based on the results presented in Table 1.

In line with our hypothesis there was no pain alarm response in the awake condition (i.e. the non-naïve subjects), indicated by stable pain ratings throughout the test-phase and non-significant model predictions (*p* = 0.216). Among participants in the two control conditions, who were naïve to noxious stimuli during the test-phase, there were significant pain alarm responses, indicated by high pain ratings during the first trials, both in the naïve_lo_ condition (*p* < 0.001) and the naïve_hi_ condition (*p* < 0.001). On average, the alarm response among naïve participants entailed pain ratings of 16 units higher than subsequent stimuli (Fig. 1). In the sleep condition, participants were unaware of the noxious stimuli prior to the test-phase and in line with our hypothesis there was a pain alarm response in the sleep condition (*p* < 0.001). In addition, an exploratory analysis of the first test stimulus reveals a significant difference between the awake and sleep condition (t(12.68) = 2.67, *p* = 0.019 Welch’s t-test). Only participants from the sleep condition that did not remember having received painful stimuli were included in the analysis.

The pain alarm response in this study was represented by an overall significant increase in pain ratings followed by a rapid return to stable pain ratings. In order to provide practically useful information for future pain studies, we estimated the number of painful stimuli needed before the pain alarm response will not confound subjective pain ratings. After being exposed to 4 stimuli, >80% of participants showed no effect, as they had less than 5 units remaining of the alarm response. Furthermore, there were large individual differences in the magnitude of the alarm response (SD = 16 units) and about 25–45% of subjects showed no such response; i.e. less than 5 units on the 0–100 pain rating scale. Individual differences are illustrated in the Supplementary Material.

## Discussion

Here we assessed the role of conscious awareness in the response to pain, by testing if noxious stimulations during sleep may lead to subsequent changes in pain ratings in the awake state. Our results indicate that a heightened reaction to novel painful stimuli—referred to as the pain alarm response—is partly dependent on conscious awareness. Participants who were fully aware of getting noxious stimuli prior to pain testing habituated to pain and did therefore not display a pain alarm response. Participants who were unaware of any noxious stimuli had a different response, as the first stimulation was rated higher than in the awake condition.

This is in line with recent evidence suggesting that pain habituation is an example of a learning process that involves both peripheral and central nervous system mechanisms^9^. In other words, even the simplest forms of pain modulation involve cognitive processes. The pain alarm response is related to habituation, yet, the term habituation refers to a lowered response after repetition of stimuli whereas the pain alarm response refers to the heightened response to initial stimuli (sometimes referred to as the “surprise effect”^8^). Previous studies have demonstrated that associative learning alters pain perception even if the conditioned stimulus is presented subliminally (i.e. outside conscious awareness)^10,11^ and functional neuroimaging data indicates greater involvement of subcortical brain regions during subliminal trials, with special emphasis on the role of the amygdala and adjacent subcortical structures known to facilitate rapid recognition of threats^12^. Yet, in this study where participants were fully asleep during pain exposures we found no significant pain learning effect. When looking closely at the pain ratings in this study (see Fig. 1) there was a small (non-significant) difference in mean pain ratings between the sleep and naïve condition. This could indicate that the pain alarm response is attenuated in sleeping participants due to some basic form of learning during sleep, yet this is speculative and needs to be studied more closely in future studies.

The necessity to always be vigilant about pain fits well with the notion that pain represents a salience detection system^2^. Saliency refers to the aspect of a stimulus that leads to increased attention if it is deemed relevant for learning and survival. The response to pain, which is a highly salient stimulus, will thus be malleable and change depending on several factors such as the sharpness^13^, novelty^14,15^ and intensity^2^ of the painful stimulus. In other words, the subsequent reaction of a painful stimulus depends on its context and how it compares to other adjacent stimuli.

Historically, habituation to has been viewed as an insignificant form of behavior where focus was initially on the physiological or behavioral part of habituation^16^. More recent studies have found evidence for a central component in pain habituation, and described it in terms of a basic physiological function that promotes a healthy balance between anti- and pro-nociceptive processes by directing attention to potentially harmful stimuli and being able to ignore them if appropriate^9^.

There is a large literature on the bidirectional relationships between sleep and pain. As many as 67–88% of patients with a chronic pain disorder also meet criteria for insomnia^17^. A recent systematic review concluded that chronic pain problems often cause fragmented insomnia like sleep disturbances^18^. Disturbed sleep, per se, is also related to a heightened pain sensitivity, both in patients suffering from insomnia^18^ as well as after in healthy populations exposed to experimental sleep deprivation^19^. A higher degree of insomnia like sleep disturbances are also related to worse pain problems in a dose-response manner^20^. In the present study, the subjects were healthy and exposed to low levels of pain so as pairing with noise could occur without interfering with sleep. While we used PSG to classify whether a subject’s sleep was disturbed by pain other paradigms, e.g. fMRI or high-density EEG, may be fruitful ways to study involved mechanisms of such associated learning.

In order to improve the chances that participants in the sleep condition would fall asleep in the lab, they were instructed to sleep a maximum of 5–6 hours per night for two nights in a row before the experiment. There was a risk that pain perception among participants in the sleep condition could be influenced by the fact that they were sleep deprived, as acute and chronic sleep deprivation may have effects on pain perception^21,22^ and lead to lower pain thresholds^22,23^. Yet, we found no difference in pain threshold between conditions and conclude that night sleep before the experiment is an unlikely confounder.

The level of intensity of the painful stimuli during sleep is an important factor to consider. In the pilot stage of this study, higher intensities of the stimuli were tested but resulted in waking the participants up. In order to minimize interfering with participants’ sleep, a lower intensity of painful stimulation was chosen. Studies on painful stimulation and how it interferes with sleep indicate that the range of stimuli that successfully can be administered without waking the participants up while still be processed by the brain is narrow^24^. For example, nociceptive stimuli have a six-fold higher probability of waking a participant up compared to auditory tones used in previous sleep studies^25,26^. Further testing with different pain modalities during sleep might be one way of investigating the boundaries of pain processing during sleep.

One limitation to this study is the high number of excluded participants from the sleep condition, where pain was administered during sleep. Participants who woke up when noxious heat was administered, or indicated a recollection of receiving pain while asleep, were excluded from our analysis leading to the possibility that our final cohort consisted of a skewed selection of individuals. Further studies will have to determine whether people who are better at sleeping during noxious stimulation are different regarding the pain alarm response, compared to individuals who wake up more easily. Furthermore, this was a nap study, hence not performing the experiment during a full night’s sleep. If the study had been using sleep during a full night instead of afternoon naps, we might not have needed the sleep deprivation. In addition, it is possible that it would have led to fewer exclusions due to longer periods of sleeping.

It is also worth to mention that the participants in the sleep condition were exposed to noxious heat while awake, during the pain calibration before the learning phase. This could have led to a reduced pain alarm response in the test phase. Furthermore, there was no randomization between the four different experiments. This could potentially have led to a difference in participant characteristics. We found no differences in baseline measurements such as pain threshold and sleepiness between groups (see Table 1), however future studies are needed using larger sample sizes.

One purpose of this study was to determine the feasibility of pain conditioning during sleep whereby all participants were subjected to a low volume sound played in the experiment room during administration of pain. We cannot rule out the possibility that this (very muted) sound influenced pain learning due to subtle distraction of participants’ attention. Nevertheless, all participants in all conditions were subject to the same procedures.

Finally, consciousness is difficult to ascertain and we cannot be certain that all participants in the sleep condition were completely unaware of noxious stimulations even if we used both EEG to verify that subjects did not wake up in combination with verbal control questions. The analyses only included subjects that did not show any awakenings during sleep when exposed to pain (based on EEG) or remembered receiving any pain after awakening. Importantly, the brain should not be seen as being entirely unconscious during sleep, but still engage in some information processing^27^. While our study investigates processing and habituation to pain during sleep, a central remaining question concern how the brain does this. A fruitful way may be to study neural correlates to painful stimuli during sleep, similar to what has been done during dreaming^28^ or in stressed subjects^29^.

To the best of our knowledge this is the first investigation of the role of conscious awareness on the pain alarm response. Results demonstrate that the pain alarm response is partly dependent on conscious awareness, as the pain alarm response is affected by knowledge of prior exposures. The results contribute with concrete suggestions on the design and interpretation of experimental studies using repeated exposures to pain, which may ultimately deepen the knowledge about the underlying mechanisms of pain perception. Also, these findings open up for a broader area of investigation where the role of consciousness in processing of noxious stimuli is determined.

## Materials and Methods

### Experimental design

Four experiments were performed, representing four experimental conditions: awake, asleep and the two control conditions naïve_hi_, naïve_lo_. In the awake and the sleep conditions, all participants received repetitions of noxious heat prior to the test-phase. The control conditions did not include any exposures to heat prior to the test-phase, which means that participants were naïve. One control condition (naïve_lo_) used the same pain intensity during the test-phase as in the awake condition. Another control condition used higher pain intensities (naïve_hi_) in order to rule out if the dynamics of the pain alarm response would differ depending on pain intensity. A different aim of this study was to test the feasibility to perform pain conditioning during sleep. Therefore, a low volume sound was played in the experiment room prior to all pain stimuli. The procedure relating to associative learning will be tested in a future study and thus not reported here. All participants provided written informed consent and the study was approved by the Regional Ethical Review Board in Stockholm (Dnr: 2015/1197-31). All experiments were performed in accordance with relevant guidelines and regulations.

### Participants

For inclusion in all four experiments, participants had to be between 18–55 years old, Swedish speaking, healthy, no medication for any chronic illness or mental disorder (however, oral contraceptive pills were allowed). Based on a structured interview, it was determined that participants in the sleep condition did not have any sleeping problems or had easily disturbed sleep. Participants in the sleep condition were required to have self-reported sleep latencies below 30 minutes, being able to sleep in the afternoon and able to fall asleep in a sleep lab (Tables 3, 4). Participants were recruited through flyers posted at the Karolinska Institute (Stockholm, Sweden) and via an advert on the website www.studentkaninen.se. Compensation for participating in any of the four studies was 400 SEK (€40). In total 114 participants were recruited (67 women, mean age 27.3 ± 8.3). Nineteen participants in the sleep condition were excluded due to remembering getting painful stimulations. Hence, only 11 participants were included in the statistical analysis (6 women, mean age 26.0 ± 9.3). One participant in the naîve_lo_ condition was excluded due to absence of pain sensation in the calibration phase. There were no excluded participants in any of the other conditions. Excluded participants in the sleep condition did not differ significantly from participants included in the analysis with respect to any of the measure regarding pain threshold (t(21.38) = 1.49, *p* = 0.15 Welch’s t-test), maximum pain (t(20.36) = −0.96, *p* = 0.346 Welch’s t-test), or sleepiness (t(14.39) = −0.008, *p* = 0.99 Welch’s t-test).

**Table 3:** Inclusion and exclusion criteria for the sleep condition.

Inclusion criteria	Exclusion criteria
Can easily fall asleep	Sleeping problems
Age 18 to 55 years	Easily disturbed by noise while sleeping
Healthy	Any medication for any chronic illness or mental disorder (however, oral contraceptive pills were allowed).
Swedish speaking	
Can sleep “anywhere”, e.g sleep lab	
Can take a nap in the afternoon	
Self-reported sleep latencies below 30 minutes

**Table 4:** Inclusion and exclusion criteria for the awake, naïve_hi_ and naïve_lo_ condition.

Inclusion criteria	Exclusion criteria
Age 18 to 55 years	Any medication for any chronic illness or mental disorder (however, oral contraceptive pills were allowed).
Healthy	
Swedish speaking	

### Materials and apparatus

Painful stimuli were administered using noxious heat via a 3 × 3 centimeter thermode (awake) or 2.5 × 5 centimeter thermode (sleep, naïve_hi_, naïve_lo_) attached to the participants’ left calf. Two comparable devices were used: in the awake condition the Medoc Pathway CHEPS model was used (Medoc Advanced Medical Systems Ltd, Ramat Yishay, Israel). In the sleep, naïve_hi_ and naïve_lo_ condition the heat stimulations were administered via the same instrument from a different manufacturer, Somedic Senselab thermotest (Somedic Senselab AB, Hörby, Sverige). The temperatures ranged from 38 to 49 degrees Celsius and each stimulus lasted for 4 seconds. Participants rated the heat stimulations verbally using a numeric rating scale (NRS) ranging from 0 (no pain) to 100 (worst imaginable pain).

The participants that were allocated to the sleep condition performed their experiment in a sleep laboratory. In order to determine if the participants were asleep during the painful stimulations in the sleep condition, EEG was used to measure sleep stages. The EEG recordings were tailored for sensitivity to sleep stages and scored by an experienced sleep researcher (PD). Sleep was verified if a participant had at least Stage 2 sleep, including sleep spindles and K-complex EEG patterns. In addition to EEG scorings, control questions were asked immediately after sleep in order to determine if a participant had any recollection of noxious stimuli during sleep. If participants were deemed consciously aware of receiving any heat stimuli during sleep they were excluded from the statistical analysis.

### Procedure

Participants were screened for inclusion and exclusion criteria and then scheduled for an experiment. After giving informed consent participants were asked to lie down on a medical bed (in the sleep condition participants lay down on a real bed in the sleep laboratory). The thermode was thereafter placed on the participants’ left calf. Ascending temperatures were applied in order to find each participant’s individual temperature that would represent “high pain”, approximately 35–45 (awake condition) or 20 (sleep condition) on a 0–100 Numeric Response Scale ranging from “no pain” to “worst imaginable pain”. The chosen “low pain” temperature was set to a fixed 3 degrees Celsius below the calibrated “high pain” temperature. The chosen combination of temperatures ranged for the ‘high pain’ stimuli between 46–49 degrees Celsius, and for the ‘low pain’ stimuli between 40–43. After the calibration in the sleep condition, the participants were instructed that they can fall asleep and that they will be exposed to a series of temperatures of the same intensity as in the calibration while asleep.

**Figure 2:**
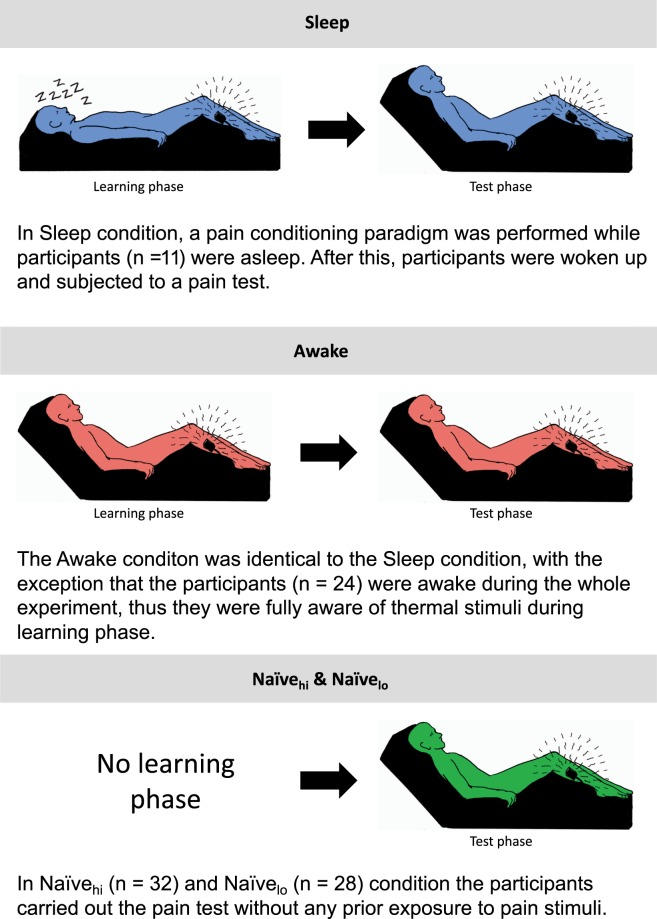
The timeline of the four experiments.

Twenty repetitions of high and low heat stimuli (corresponding to the “high pain” and “low pain” temperatures) were given in the awake condition and a maximum of forty stimuli were given in the sleep condition. The stimulations were administered according to a predetermined schedule that was the same both for the awake and sleep condition. The amount of time between each heat stimulation was randomized. At least 25 seconds passed between each stimulation to avoid sensitization. No more than 50 seconds passed between two heat stimulations. In the sleep condition, the stimulations started once the participant had been in stage two sleep for at least twenty minutes. The aim was to administer stimulations during stage two sleep, however, due to the time constraints with a nap study, the stimulations were also administered during stage three and REM-sleep. If participants woke up in the sleep condition the stimulation schedule was paused until they reached stage two sleep again. After this initial exposure to pain, where participants were passively just receiving the stimulation without giving any ratings, there was a pause of approximately 5–10 minutes before the test-phase. Participants were now instructed they were about to receive a series of heat stimulations and were asked to rate the temperatures from 0–100 NRS. The test-phase included 9 trials of a temperature that was in the middle of each participant’s calibrated high and the low temperature. Two non-painful temperatures were administered prior to the test-phase as a warm-up. After each trial the participants were asked to rate their perceived pain intensity (Fig. 2).

Some procedures were unique for participants in the sleep condition: The first six participants were asked to shorten their sleep with at least two hours one night prior to the experiment, with a maximum of sleep of 6 hours (mean hours slept 5.25 ± 0.69). Due to participants waking up during the experiment, the rest of the participants were asked to sleep maximum 5 hours per night for two nights before the experiment (mean hours slept per night 4.83 ± 0.46).

After giving informed consent, the EEG equipment was attached to the participant’s head. Before the exposures of noxious stimuli could start, participants were instructed they could now go to sleep. All participants in all four experiments were debriefed about the full purpose and potential findings of the study at the end of the experiment.

### Data reduction and statistical analyses

To estimate the rate of decline (i.e. negative growth) of the alarm response (g), a mixed effects exponential growth curve model was applied to data, with the number of prior stimuli as the independent variable (x) and observed ratings of pain as the dependent variable (y). Two latent variables (η, ξ) were modeled as random effects to account for individual differences in the asymptote (a), which represents the latent pain experience after the alarm response has declined to zero, and the alarm response itself (r):

$${y}_{ij}=a+{\eta }_{j}+(r+{\xi }_{j}){e}^{g{x}_{ij}}+{{\epsilon }}_{ij}$$

where:

$$\begin{array}{c}\eta \sim N(0,{\sigma }_{\eta }^{2})\\ \xi \sim N(0,{\sigma }_{\xi }^{2})\\ {\epsilon }\sim N(0,{\sigma }_{{\epsilon }}^{2})\end{array}$$

The model fitted on data included parameters to estimate the asymptote (a) and the alarm response (r) separately for each of the four groups, but the rate of the decline (g) was kept constant across all groups. Exploratory analysis of group differences between the awake condition and sleep condition on the first test stimulus was calculated with Welch Two Sample t-test. Differences in baseline pain sensitivity and age between groups were analyzed with Welch’s ANOVA.

Data was analyzed using *R* (version 3.5.1, R Core Team, 2017), applying the procedure *nlmer* from the package *lme4*^30^ to estimate the model. The package *nlWaldTest*^31^ was used to calculate group specific estimates. SPSS (version 24) was used to estimate differences in baseline pain sensitivity.

The datasets generated during and analyzed during the current study are available from the corresponding author on reasonable request.

## Supplementary information

Supplementary information
