# Supplementary material for: The pain alarm response - an example of how conscious awareness shapes pain perception
Source: Sci Rep. 2019 Aug 28;9:12478. doi: 10.1038/s41598-019-48903-w (PMC6713713; doi:10.1038/s41598-019-48903-w)
Supplement: Supplementary file 1 — Supplementary information [file 41598_2019_48903_MOESM1_ESM.pdf]

The pain alarm response - an example of how conscious awareness shapes pain perception

Moa Pontén*, Jens Fust, Paolo D'Onofrio, Rick van Dorp, Linda Sunnergård, Michael Ingre, John Axelsson, Karin Jensen

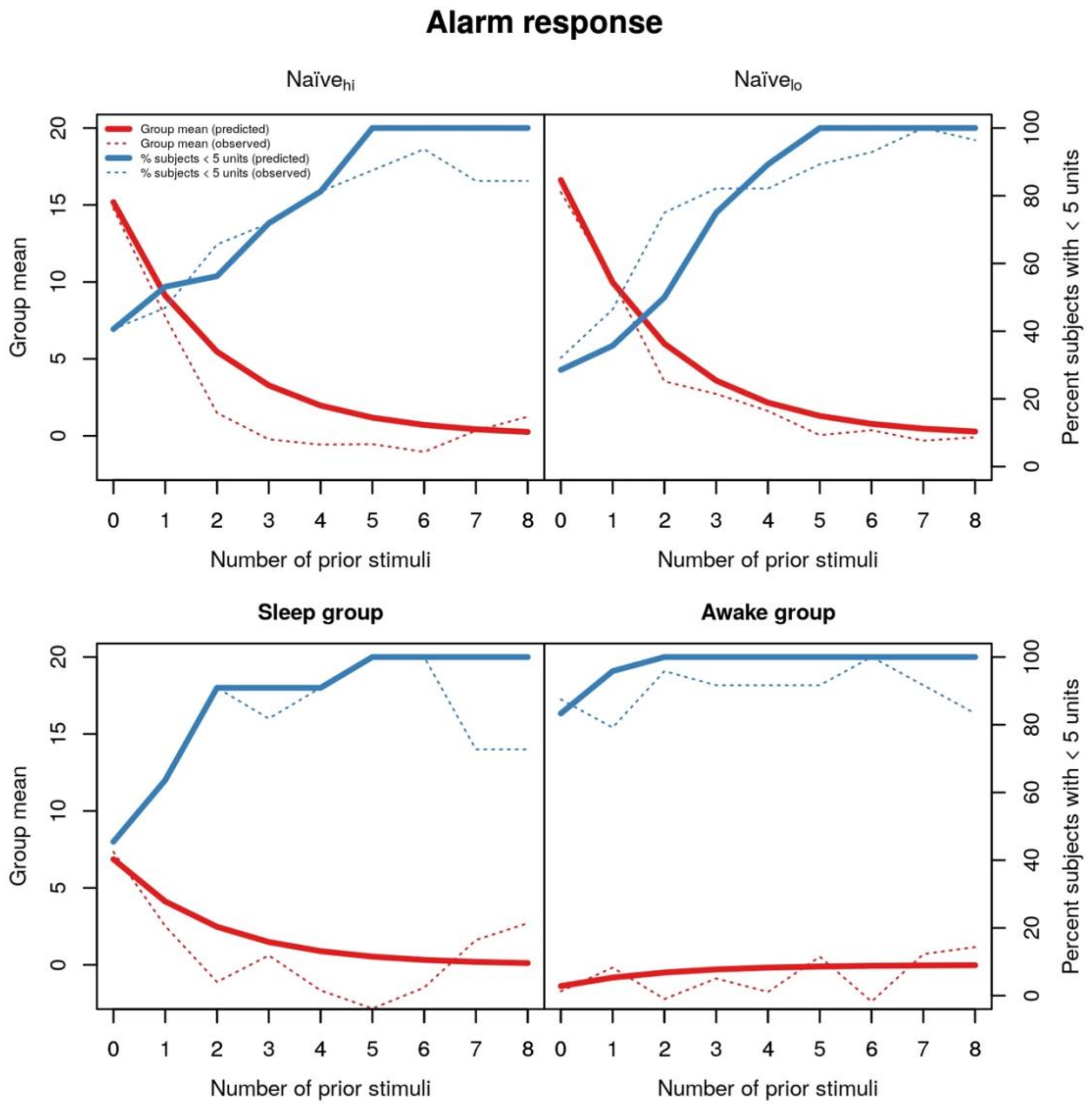

Supplementary figure 1. The magnitude of the alarm response and percentage of participants with an alarm response (< 5 NRS) during the test phase in the four different conditions.

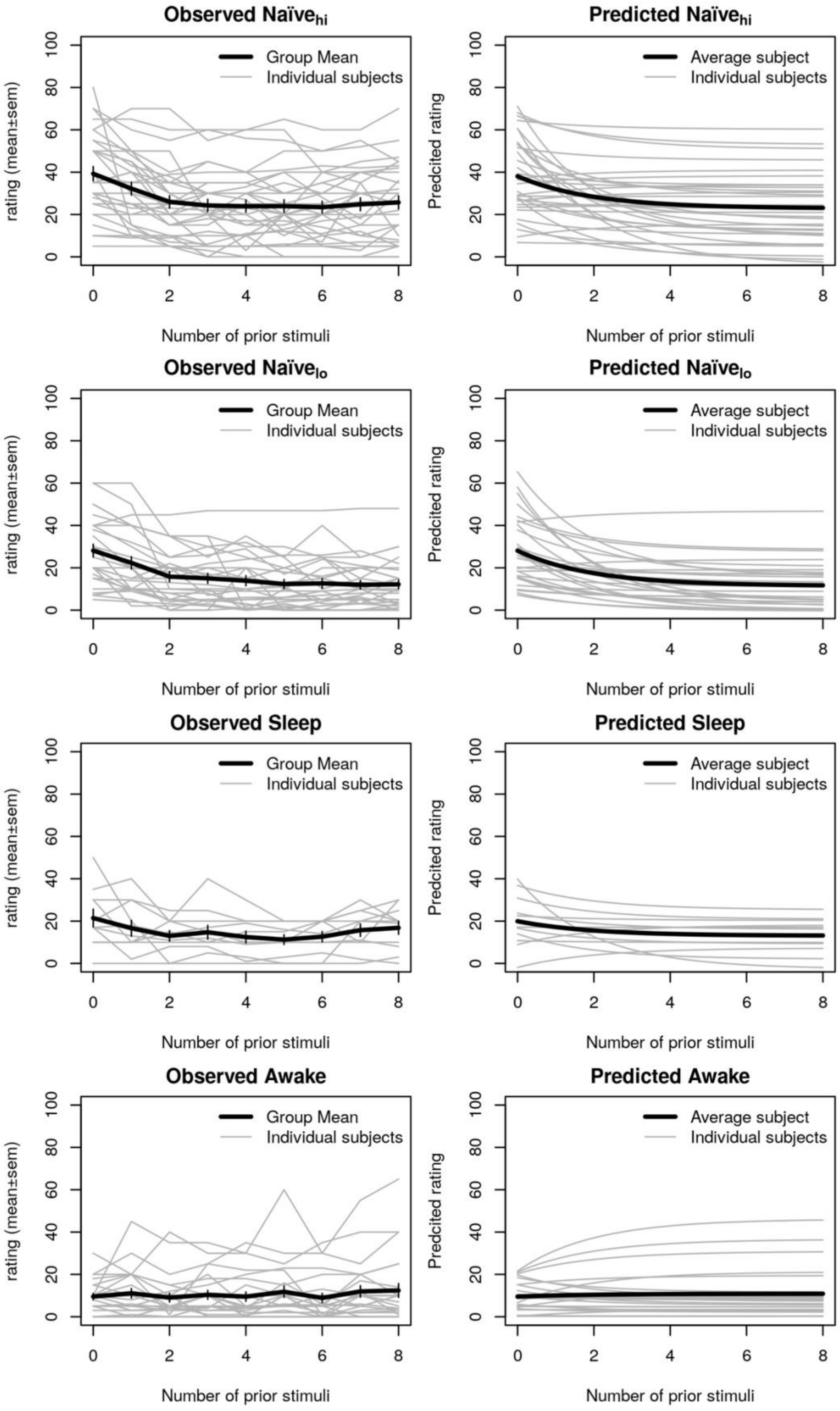

Supplementary figure 2. The observed and predicted ratings in the four different conditions.
